# Two heterozygous mutations in the calcium/calmodulin‐dependent serine protein kinase gene (*CASK*) in cases with developmental disorders

**DOI:** 10.1002/mgg3.2065

**Published:** 2022-09-28

**Authors:** Kunfang Yang, Longlong Lin, Fang Yuan, Xiaoguang Li, Zhiping Liu, Xiaoping Lan, Yilin Wang, Yun Ren, Jiaoyan Li, Yucai Chen

**Affiliations:** ^1^ Department of Neurology, Shanghai Children's Hospital, School of Medicine Shanghai Jiao Tong University Shanghai China; ^2^ Department of Emergency Shanghai United Family Hospital Shanghai China; ^3^ Department of Pediatrics Shanghai United Family Hospital Shanghai China

**Keywords:** *CASK*, developmental disorder, Han Chinese, microcephaly, X‐chromosome inactivation

## Abstract

**Background:**

The calcium/calmodulin‐dependent serine protein kinase gene (*CASK*) is an essential gene in mammals, critical for neurodevelopment. The purpose of this study is to expand the understanding of the diagnosis of *CASK*‐linked disorders.

**Materials/Methods:**

From clinical and genetic mutational analyses, relevant data in 2 Han Chinese patients were collected and analyzed. Real‐time quantitative PCR (RT‐qPCR) was performed to investigate the *CASK* expression levels in the patients. The X‐chromosome inactivation (XCI) patterns of the patients and their nuclear families were tested by quantitation of methylation of the polymorphic human androgen receptor (HUMARA) locus.

**Results:**

Two Han Chinese patients both presented with intellectual disability (ID), microcephaly with pontine and cerebellar hypoplasia (MICPCH). Two de novo mutations of c.82C>T (p.Arg28*) and c.846C>G (p.Tyr282*) in *CASK* have been investigated and predicted to be deleterious, which have produced truncated proteins. The functional protein association network of STRING (http://string‐db.org) generated three‐dimensional (3D) atomic models based on protein sequences in *CASK* and two Arg28 and Tyr282 residues were marked. RT‐qPCR showed lower copy numbers of *CASK* expression in the patients than in their parents, as well as the sex‐ and age‐ matched control groups. Patient 1 showed a skewed XCI pattern, while no related changes noted in patient 2.

**Conclusions:**

Patients carrying different nonsense variants may have different degrees of different clinical phenotypes. This study expands the spectrum of genotype and phenotype correlations of *CASK*‐linked disorders in the Han Chinese ethnicity and provides new insights into the molecular mechanism.

## Graphical abstract

Patients carrying different nonsynonymous variants had different clinical phenotypes. This study expands the spectrum of genotype and phenotype correlations of CASK disorders in the Han Chinese ethnicity, provides new insights into the molecular mechanism.

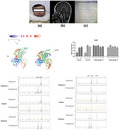

## 1 BACKGROUND

The calcium/calmodulin‐dependent serine protein kinase gene (*CASK*) (NCBI Reference Sequence: NM_001126054.2) is an essential gene in mammals, critical for neurodevelopment. Mutations are associated with different neurological disorders, resulting in phenotypes that range from intellectual disability to lethality, manifested as intellectual disability (ID), microcephaly with pontine and cerebellar hypoplasia (MICPCH), epileptic encephalopathy, X‐linked ID (XLID) with or without nystagmus, and autistic spectrum disorder (ASD) (Lee et al., 2020; Popp et al., 2017; Seto et al., 2017). MICPCH and XLID are its main types of manifestation.

The CASK protein includes a Ca^2+^/calmodulin‐dependent kinase domain, two L27 domains (from Lin‐2, Lin‐7), a postsynaptic density‐9/discs large/zonula occludens‐1 (PDZ) domain, an src homology 3 (SH3) domain and a guanylate kinase domain (Hata et al., 1996). Through these protein domains, the CASK proteins are able to interact with other proteins.

In addition, they are mostly present in dendrites, axons, synapses, and the nuclei of neurons, wherein they control several cellular functions, including the formation of the dendritic spine, outgrowth and differentiation of axon, transcriptional control, and cellular metabolism (Kuo et al., 2010; Mukherjee et al., 2020; Pan et al., 2021; Patel et al., 2020; Srivastava et al., 2016). Expression is significantly high in the central nervous system (CNS), especially during the period of brain development. It can transmit to the nucleus and interact directly with transcriptional factors responsible for conventional brain structure formation (Murakami et al., 2019).

The diagnosis of a *CASK* disorder is based on a female who is heterozygous or on a male who is hemizygous for a *CASK* pathogenic variant (Moog & Kutsche, 2013). Many mutations in *CASK* were reported to lead to *CASK*‐linked disorders (Hayashi et al., 2017; Huang & Hsueh, 2009; Studtmann et al., 2019), while only few cases of *CASK*‐linked disorders have been reported in Han Chinese. We recently encountered two cases of females whose clinical characteristics were consistent with those of *CASK*‐linked disorders. By next‐generation sequencing (NGS), two de novo heterozygous mutations in *CASK* were detected. Herein, we present the clinical characteristics of these two patients and explore the possible underlying pathogenesis of the disease.

## 2 MATERIALS AND METHODS

### 2.1 Patients

Two Han Chinese patients were diagnosed with *CASK*‐linked disorders at the pediatric neurology clinic of the Shanghai Children's Hospital from 2019 to 2021. Both cases fulfilled the criteria for the diagnosis of *CASK*‐linked disorders. Family histories were negative for neurological disorders. Screening for inherited metabolic disorders were insignificant as well for both cases and their perinatal and neonatal courses were unremarkable.

### 2.2 Variation detection

After admission, whole blood samples were obtained from the patients and their parents. NGS was performed according to standard procedures (Yang, Cheng, et al., 2018; Yang, Yin, et al., 2018). Sequencing genomic DNA samples were sonicated, followed by hybridization with the NimbleGen 2.0 probe sequence capture array (Roche, Shanghai, China) to enrich the exon DNA (Joy Orient, China). By qPCR, libraries were first tested for enrichment and size distribution. The concentration was tested using the Agilent Bioanalyzer 2100. The samples were sequenced on the sequencer (Hiseq2500, Illumina, California, USA). Each sample was performed for two parallel reactions. Data filtering, mapping, and variant detection were applied. Exon‐enriched DNA was sequenced according to the manufacturer's instruction.

The raw image files were processed using the BclToFastq (Illumina, California, USA) for base calling and producing the raw data. Low‐quality variations were filtered out using the quality score of Q20. The sequencing reads were aligned to the NCBI human reference genome (hg19) using Burrows‐Wheeler Aligner (BWA). Samtools and Pindel were applied for analyzing the single‐nucleotide polymorphism (SNP) and sequence indexing.

Synonymous changes and SNPs with the minor allele frequency (MAF) higher than 5% were removed; nonsynonymous changes were filtered using SIFT software. The functional mutations and their relationship to *CASK*‐linked disorders were further analyzed. STRING server (http://string‐db.org) was used for functional enrichment analysis.

### 2.3 QPCR analysis for transcription levels of *CASK*

Total RNA from the patients and their parents were extracted by using a QIAGEN RNA Preparation Kit (QIAGEN Inc., CO, Germany) (Wang et al., 2020). The cDNA from these samples was used by reverse transcription and synthesized by PrimeScript™ Strand cDNA Synthesis Kit/RT Master Mix (Takara Shuzo Co., Ltd., Japan). The qPCR primers used to test the expression of *CASK* in the venous blood as follows: forward primer, 5′‐TGTAGCTGGAGGACGTGTTG‐3′ and reverse primer, 5′‐AGACGTCTACAGGCTTTCCG‐3′. Real‐time qPCR was done by using an SYBR Premix Ex Taq II in a LightCycler® 96 Instrument (Roche, AG Schweiz). The relative expressions of *CASK* in patients' nuclear family were investigated, as well as in sex‐ and age‐matched control groups. The data were expressed as mean ± *SD* of triplicate experiments (**p < .05, ***p < .01 vs. control group).

### 2.4 XCI analysis based on human androgen receptor ( *AR* ) gene polymorphism

Inactivation patterns of the X‐chromosome of the patients and their nuclear families were tested by a methylation‐based analysis on the human *AR* using a reported protocol (Cutler Allen et al., 1992; Kassim et al., 2004). Briefly, 200 ng of DNA from peripheral venous blood cells was digested with *Hpa*II (New England Biolabs, USA) at 37°C overnight, followed by enzymatic inactivation. Both digested and nondigested DNA samples were used as a pattern to amplify the *AR* to control the quality. PCR amplification on both digested and undigested DNA was performed using primers specific to the methylation regions of the short tandem repeats of exon1 in *AR*. The fluorescence‐labeled PCR products were denatured at 95°C (5 min) and further analyzed by capillary gel electrophoresis using Image Lab software. Analysis was performed with Sub‐Cell GT Agarose Gel Electrophoresis Systems (Bio‐Rad Laboratories, Hercules, CA, USA).

## 3 RESULTS

### 3.1 Clinical manifestations of the patients

Patient 1 was examined in our clinic at the age of 6 months. She exhibited psychomotor delay with difficulty in holding head up, rolling over, sitting with support, reaching for toys, raking hands, and passing objects hand to hand. She did not turn to voice. Her head circumference was 36 cm which is on the below 3% percentile. On further examination, there was mild hypotonia. There was no nystagmus, special facial abnormalities, high arched‐palate, or hypohidrosis.

Brain magnetic resonance imaging (MRI) showed decreased volume of the bilateral cerebellar hemispheres and brain stem. Cistern was wide with bilaterally widened temporal intracranial space. On Gesell intelligence test (July 31, 2020): adaptability 45 points, big movement 42 points, fine movement 50 points, language 61 points, personal social 40 points; Peabody assessment: equivalent to the age of 1 month, GMQ 53, FMQ 52, TMP 48. BAEP: the differentiation of the left III/V wave was poor, the amplitude was flat, and the right side was normal. The electroencephalogram (EEG) was unremarkable. Her parents are unaffected.

**FIGURE 1:**
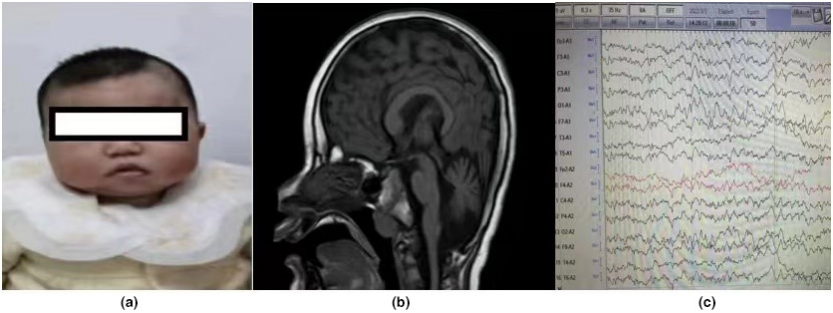
(a–c) The representive clinical characteristics of patient2: (a) Microcephaly with wide‐set eyes, wide nasal bridge, small nose andhigh‐arched palate. (b) Decreased volume of the bilateral cerebellarhemispheres and brain stem decreased on brain MRI. (c) EEG unremarkable.

Patient 2 was seen at the clinic at the age of 13 months. She was a product of in‐vitro fertilization. She presented with psychomotor developmental delay with difficulty in sitting independently, standing with support, crawling, pincer grasping, pointing, and looking for dropped or hidden objects. Her language development was also delayed. No words were formed even “Mama”, “Baba”, or “Dada.”. She also had feeding problems. On examination, there was mildly increased muscle tone with hyperreflexia. Microcephaly was noted. No nystagmus but with wide‐set eyes, wide nasal bridge, small nose, and high‐arched palate (Figure 1a). Hypohidrosis was observed.

Brain MRI showed that the volume of the bilateral cerebellar hemispheres and brain stem were decreased (Figure 1b). EEG was unremarkable (Figure 1c). Her parents are unaffected.

### 3.2 Gene sequencing analysis

Gene mutation analysis of patient 1 revealed a de novo nonsense mutation of c.82C>T (p.Arg28*) in *CASK*, resulting in a stop codon in a heterozygous‐like pattern. This mutation was not detected in the proband's parents (Figure S1). The locus 28 showed highly conserved by sequence conservation analysis (Figure S2). The other mutation carriers or variants with unknown pathogenic significance were detected (Table 1).

**TABLE 1:** List of mutation carriers or variants with unknown pathogenic significance

Gene	OMIM	Inheritance	HG19 position	Transcript	Nucleotide change	Zygous state	Source
*AARS1*	601065	AD /AR	chr16: 70288599	NM_001605	c.2324_2325del (p.S775Cfs*22)	Heterozygous	Mother (heterozygous)
*BCL11B*	606558	AD	chr14: 99642394	NM_138576	c.779C>T (p.T260M)	Heterozygous	Father (heterozygous)
*BRPF1*	602410	AD	chr3: 9785474	NM_001003694	c.2524C>T (p.R842W)	Heterozygous	Mother (heterozygous)
*KCNB1*	600397	AD	chr20: 47990392	NM_004975	c.1705G>C (p.V569L)	Heterozygous	Father (heterozygous)
*PHACTR1*	608723	AD	chr6: 13283783	NM_030948	c.1639G>T (p.A547S)	Heterozygous	Father (heterozygous)
*PHF21A*	618725	AD	chr11: 45967536	NM_001101802	c.1304A>G (p.N435S)	Heterozygous	Mother (heterozygous)
*PRMT7*	610087	AR	chr16: 68380059	NM_019023	c.1067A>C (p.N356T)	Heterozygous	De novo
*SLC36A2*	608331	AD /AR	chr5: 150726957	NM_181776	c.65C>A (p.S22*)	Heterozygous	Father (heterozygous)

Patient 2 had a de novo heterozygous nonsense mutation of c.846C>G (p.Tyr282*) in *CASK*, resulting in a stop codon in a heterozygous‐like pattern. This mutation was not detected in the proband's parents (Figure S1).

### 3.3 STRING server for the functional enrichment analysis of nonsense mutations

**FIGURE 2:**
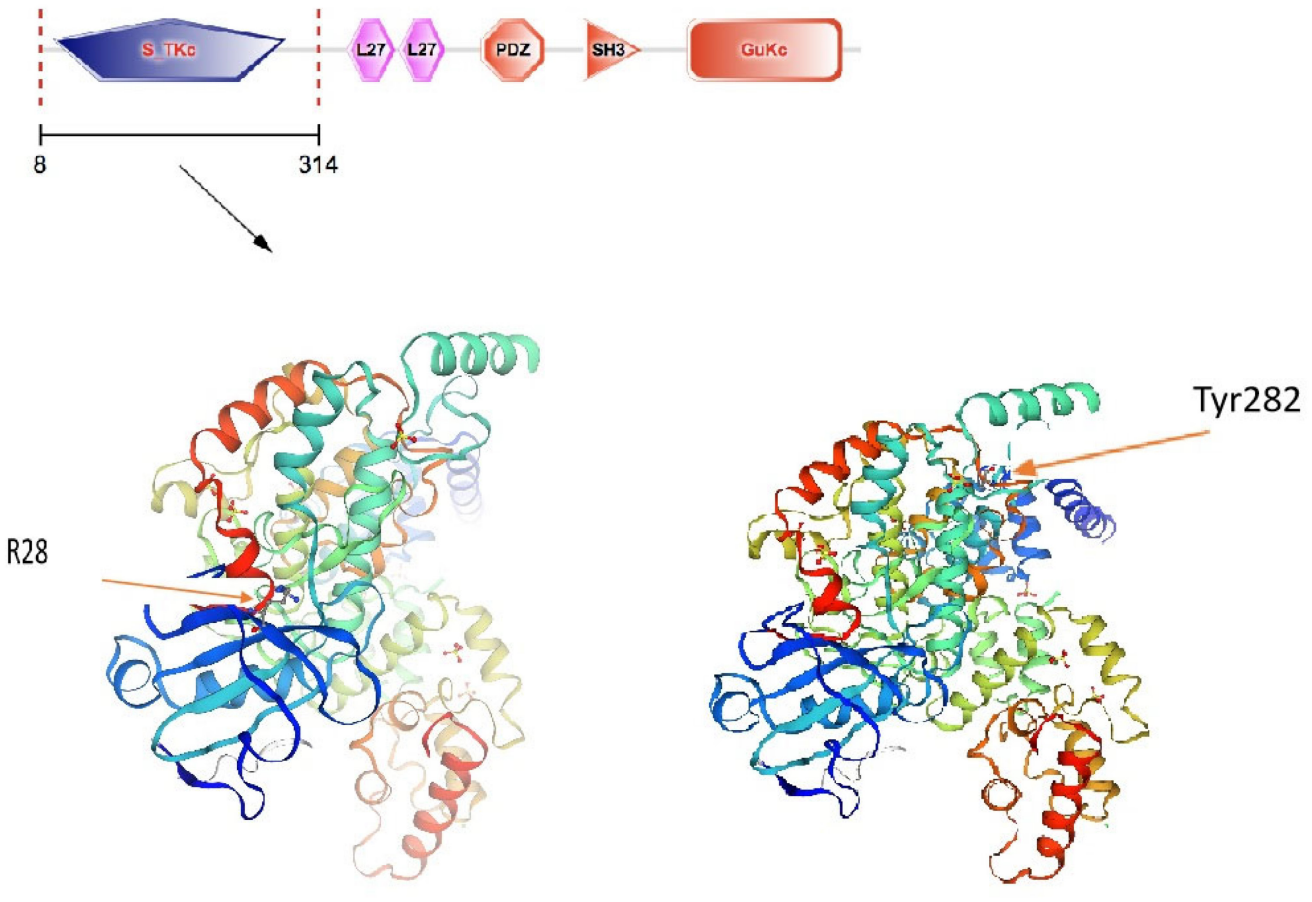
Predict 3D structure of CaMKII domain of CASK protein. Two Arg28 and Tyr282 residues were marked.

STRING server‐generated 3D atomic models based on protein sequences in *CASK*. Two Arg28 and Tyr282 residues were marked (Figure 2). They were both in Ca^2+^/calmodulin‐dependent protein kinase II (CaMKII) domain and all the downstream domains (two L27 domains, a PDZ domain, an SH3 domain, and a guanylate kinase domain) can be affected.

### 3.4 The transcription levels of *CASK* in the patients and in the control group

**FIGURE 3:**
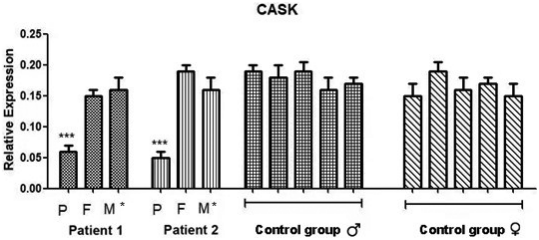
The relative expressions of *CASK* were investigated with RT‐qPCR in the patients' nuclear families, as well as in sex‐ and age‐matched control groups. The patients exhibited significantly lower expressions of the *CASK* compared to those of their parents (*P: Patient, F: Father, M: Mother). The discrepancy in the levels of *CASK* was not related to age or sex according to the results of the control groups. The data were expressed as mean ± *SD* of triplicate experiments (***p < .01 vs. control group).

RT‐qPCR with the same primer sets confirmed decreased copy numbers of *CASK* expression in patients than their parents, as well as in sex‐ and age‐matched control groups (five males and five females). The discrepancy in the levels of *CASK* was not related to age or sex according to the results of the control groups (Figure 3).

### 3.5 Study skewed XCI patterns in the patients

**FIGURE 4:**
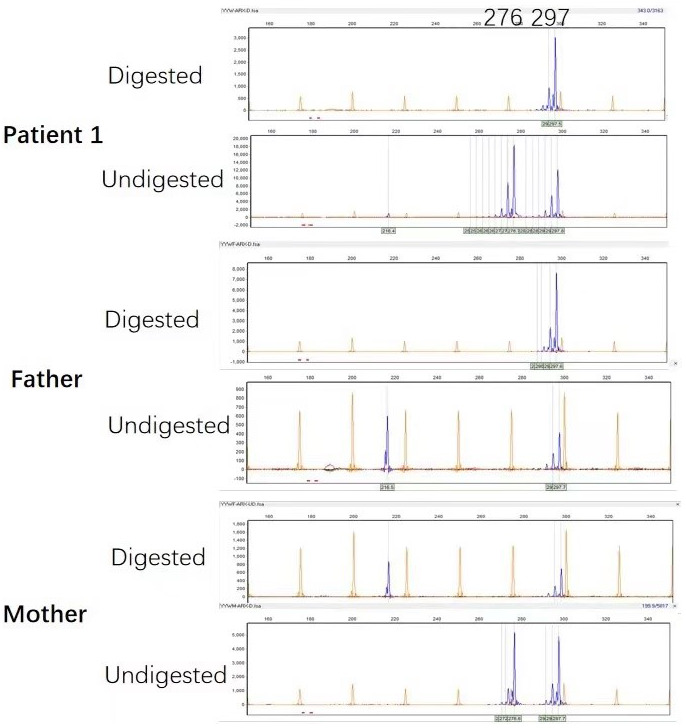
Patient 1's PCR products of the *AR* with and without *Hpa*II digestion of the nuclear family. The peak represents the amplified *AR* allele. The PCR products of the patient derived from the undigested DNA yielded two peaks because of the different numbers of CAG repeats in the two alleles (276 bp from the mother and 297 bp from the father). However, after *Hpa*II was digested, one peak (297 bp) appeared hypermethylated and the other peak (276 bp) was almost completely missing. The results revealed a skewed XCI pattern in the patient with the paternal allele highly inactivated.

**FIGURE 5:**
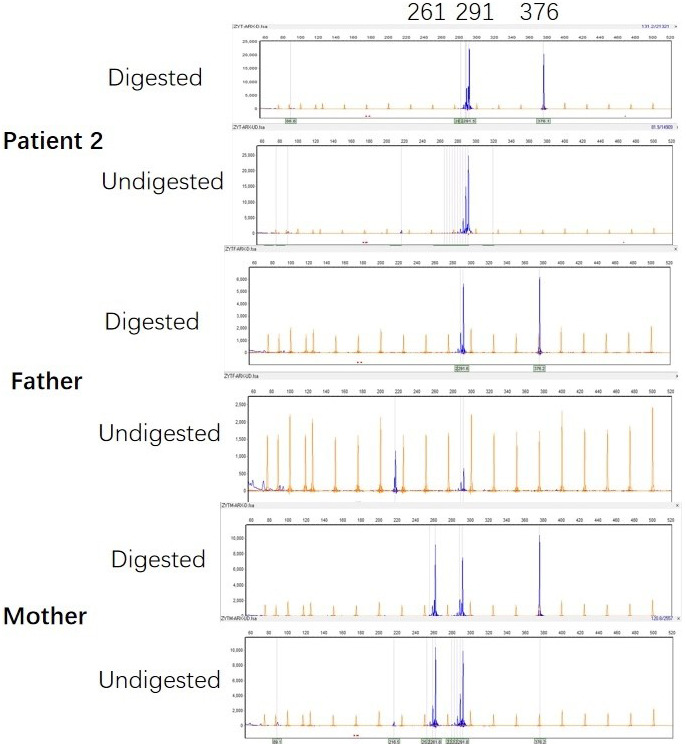
Patient 2's PCR products of the *AR* with and without *Hpa*II digestion of the nuclear family. The peak represents the amplified *AR* allele. PCR products derived from the undigested DNA yielded one peak of 291 bp, the same length of the chromosomes, respectively, inherited from the parents. The *AR* polymorphism analysis did not show XCI pattern.

Microsatellite PCR products of *AR* with or without digestion showed that the number of repeats within *AR* determines the size of the allele. In patient 1, PCR products derived from the undigested DNA yielded two peaks because of the different numbers of CAG repeats of the two alleles, 276 bp from the mother and 297 bp from the father (Figure 4). The X‐inactivation pattern was thought to be skewed if the proportion of the two alleles after digestion was at least 20:80 (Giorgio et al., 2017). The experiment showed that the patient had a skewed XCI pattern (13%:87%) with the paternal allele remarkably inactivated. In patient 2, PCR products derived from the undigested DNA yielded one peak of 291 bp, the same length of the chromosomes, respectively, inherited from parents (Figure 5). The *AR* polymorphism analysis did not show XCI pattern.

## 4 DISCUSSION

CASK domain arrangement is considered to be conserved in all metazoans, and individual domain structures present a high degree of evolutionary conservation (LaConte et al., 2014). Hence, mutations in *CASK* may cause pathogenesis via several mechanisms. There are several different computational programs available to predict or present the protein structures for mutated genes, STRING server was used in this study to present the affected protein domains based on 3D structural information. Identifying a point mutation interrupting a special protein interaction would be useful in dissecting its molecular function. In our study, two probands showed two nonsense mutations, resulting in two stop codons, which can affect all proteins in downstream domains (Figure 2). Although discovering mutations related to a particular disorder is informative, it is only the first step. Only after the characteristic of each mutation is identified, any potential therapeutic methods can be explored.

With the increase of family or sporadically affected females reported in variable clinical features, the clinical spectrum of *CASK* disorder is wider than previously reported. But the mechanism of pathogenesis in females has rarely been fully elucidated. Toshiyuki Seto et al. (2017) reported a novel *CASK* mutation identified in siblings, who exhibited different clinical manifestations due to different XCI patterns.

In our study, patient 1 had a de novo nonsense mutation of c.82C>T (p.Arg28*) in *CASK*. The codon coding arginine was substituted by a stop codon. The STRING server showed the mutated CASK protein was in CAMKII domain and all the downstream domains can be affected. RT‐ qPCR showed significantly lower levels of *CASK* expression than their parents. The discrepancy was not related to age or sex under the comparison with the sex‐ and age‐matched control groups, which indicated that the lower levels of *CASK* expression might be related to the nonsense mutation instead of other factors. Since *CASK*‐related syndromes exhibit X‐linked inheritance, female carriers may be unaffected or present with mild clinical manifestations. While patient 1 exhibited obvious clinical features of *CASK* disorder, we predicted that the possible hypothesis was related to the XCI pattern by methylation. By methylation‐sensitive PCR and *AR* CAG repeat polymorphism analysis, this patient showed a skewed XCI pattern. Hence we hypothesized that this patient's spontaneously mutated gene was on the activated X‐chromosome, which was unable to produce sufficient levels of *CASK*, resulting in a series of clinical manifestations. In a word, according to the clinical characteristics, patient 1 belonged to a syndrome of ID and MICPCH, with significantly lower levels of *CASK* expression, and XCI mechanism may exist.

Patient 2 had a de novo nonsense mutation of c.846C>G (p.Tyr282*) in *CASK*, which was not inherited from her parents. The codon coding tyrosine was substituted by a stop codon. STRING server showed the mutated CASK protein was in CAMKII domain and all the downstream domains can be affected. RT‐ qPCR showed significantly lower levels of *CASK* expression than their parents, as well as the sex‐ and age‐matched control groups. She did not show XCI pattern by *AR* polymorphism methylation‐based analysis. We hypothesized this patient may have other possible mechanisms like nonsense‐mediated mRNA decay (NMD) which can also lead to a reduction in *CASK*. NMD was a processing pathway in cells. Its mechanism prevents the production of potentially toxic truncated proteins by recognizing and degrading transcripts containing premature translation‐termination codon (PTC). Like a broom, it clears the wrong RNA to prevent its generative transformation into abnormal proteins, which leads to lower levels of *CASK* expression in this patient. The confirmed mechanism remains unclear. NMD mechanism or other further hypothesis need to be investigated. According to the clinical characteristics, patient 2 also belonged to a syndrome of ID and MICPCH.

MRI in patients with *CASK* mutations showed normal size of the corpus callosum, but with a reduced size of the cerebrum, midbrain, cerebellar vermis, or hemispheres (Takanashi et al., 2010). Both of our detected patients showed microcephaly with bilateral cerebellar hemispheres and brain stem decreased volume, which was considered to be relevant to the disruption of the CASK‐neurexin interaction (LaConte et al., 2018). The features of delayed development and brain images together with mild facial abnormality are strongly suggestive of this disorder. Prompt subsequent testing of *CASK* should be suggested (Moog et al., 2011).

*CASK*‐linked disorders are inherited in an X‐linked manner. Loss‐of‐function of *CASK* usually leads to the manifestation of *CASK*‐linked disorders in females. In both cases, the parents were examined and found to be without *CASK* mutation or neurological symptoms. Mutations in *CASK* are usually “de novo” and they affect more women than men (Rivas et al., 2017). As in our clinical cases, both are girls, in whom “de novo” mutation were confirmed while we could not see related changes in their parents.

## 5 CONCLUSIONS

In summary, patient 1, with a nonsense mutation of c.82C>T (p.Arg28*) in *CASK*, had early psychomotor delay, and MICPCH, without nystagmus, special facial abnormalities, epileptic encephalopathy, or ASD. Patient 2, with a nonsense mutation of c.846C>G (p.Tyr282*) in *CASK*, had early psychomotor developmental delay, mild facial abnormalities and MICPCH, without nystagmus, epileptic encephalopathy, or ASD. Owning to the identification of *CASK* mutations in patients with different clinical features, the clinical entity of *CASK* disorder is expanding. Our preliminary research put forward the possible mechanisms underlying the pathogenesis of our patients. The speculation of whether the patient 1's spontaneously mutated *CASK* was on the activated X‐chromosome and the possible NMD mechanism for patient 2's low levels of *CASK* expression remained to be further confirmed. The induction of patients' naturally formed somatic cells into stem cells would be a valuable way for further research.

In conclusion, two patients both presented with ID, microcephaly, and MICPCH, with mild clinical differences (with or without special facial abnormality), which expands the spectrum of genotype and phenotype correlations of *CASK*‐linked disorders in Han Chinese, providing new insights into the molecular mechanism of *CASK*‐linked disorders and helping to further explore the contribution of other protein–protein interactions. Due to the limited small number of patients, further large‐scale case studies are needed to investigate the genotype–phenotype correlations and specific treatment approaches of *CASK*‐linked disorders (Becker et al., 2020).

## CONFLICT OF INTEREST

None.

## AUTHOR CONTRIBUTIONS

*StudyDesign*: Kunfang Yang, Fang Yuan, Zhiping Liu, Yun Ren and Yucai Chen. *Data Collection*: Kunfang Yang, Longlong Lin, Xiaoping Lan and Yun Ren. *Statistical Analysis*: Kunfang Yang, Longlong Lin, Fang Yuan, Xiaoping Lan. *Data Interpretation*: Longlong Lin and Jiaoyan Li. *ManuscriptPreparation*: Kunfang Yang, Xiaoguang Li, Zhiping Liu and Yilin Wang. *Literature Search*: Xiaoguang Li, Yilin Wang and Yucai Chen. *Funds Collection*: Yucai Chen.

## ETHICAL COMPLIANCE

The parents of the probands were informed by the consultant in the Department of Pediatric Neurology about the purpose of the DNA analysis. The samples were investigated after obtaining informed consent from the parents, as well as the legal representatives of the sex‐ and age‐matched control groups. This work was approved by the Ethics Committee of Shanghai Children's Hospital.

## Supporting information


Supinfo

## Data Availability

All datasets generated for this study are included in the Supplementary Materials.
