# Supplementary material for: Two heterozygous mutations in the calcium/calmodulin‐dependent serine protein kinase gene (CASK) in cases with developmental disorders
Source: Mol Genet Genomic Med. 2022 Sep 28;10(11):e2065. doi: 10.1002/mgg3.2065 (PMC9651610; doi:10.1002/mgg3.2065)
Supplement: Supplementary file 1 — Supinfo [file MGG3-10-e2065-s001.docx]

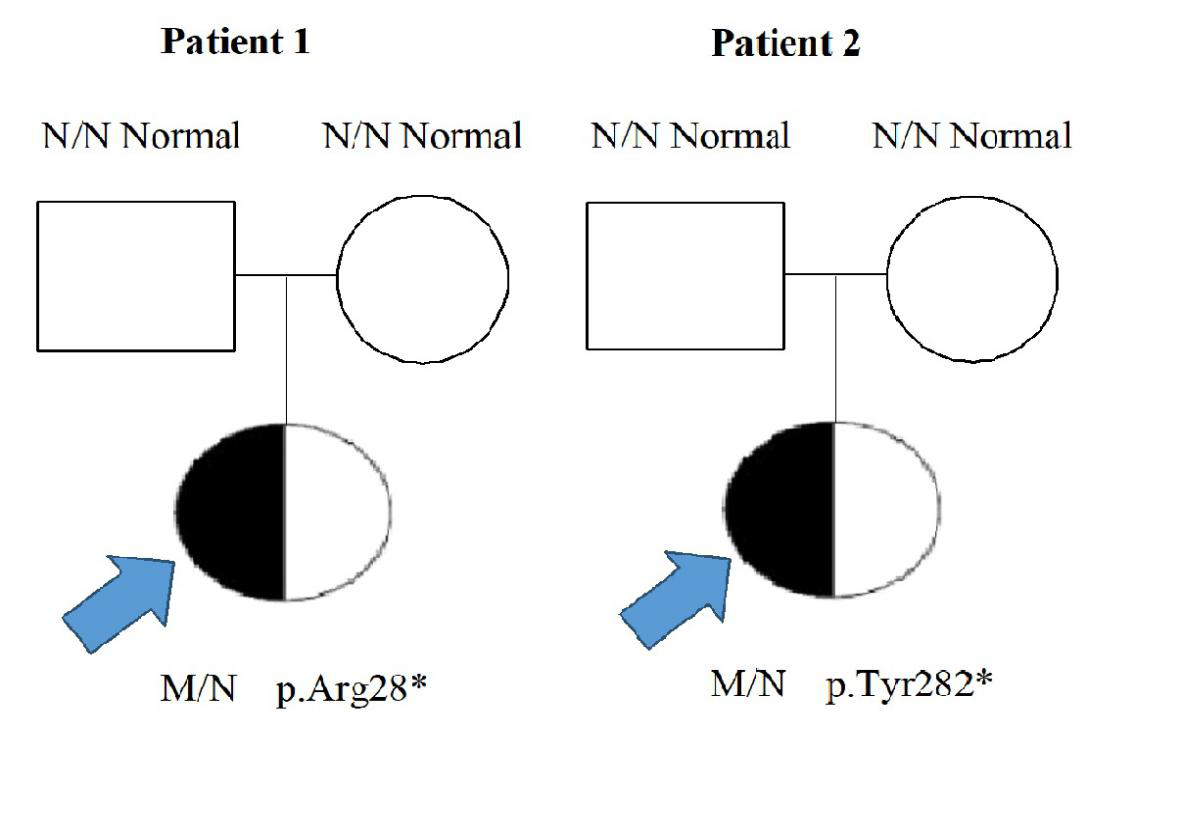


**Supplementary Figure 1** Gene sequencing analysis


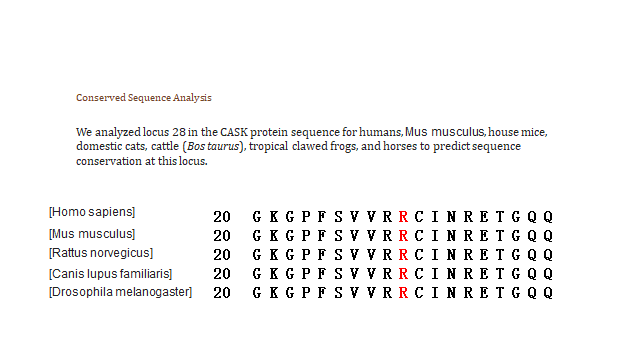


**Supplementary Figure 2** Sequence conservation analysis of locus 28 in *CASK*
